# Epidemic characteristics of hemorrhagic fever with renal syndrome in China, 2006–2012

**DOI:** 10.1186/1471-2334-14-384

**Published:** 2014-07-11

**Authors:** Shuo Zhang, Shiwen Wang, Wenwu Yin, Mifang Liang, Jiandong Li, Quanfu Zhang, Zijian Feng, Dexin Li

**Affiliations:** 1 Key Laboratory of Medical Virology, NHFPC, National Institute for Viral Disease Control and Prevention, China CDC, 155 Changbai Road, Beijing, 102206, China; 2 Chinese Center for Disease Control and Prevention, Beijing, China

**Keywords:** Hemorrhagic fever with renal syndrome (HFRS), Hantaviruses, Epidemics, Phylogenetic analysis

## Abstract

**Background:**

Hemorrhagic fever with renal syndrome (HFRS) caused by hantaviruses is a serious public health problem in China. The National Notifiable Disease Surveillance System (NNDSS) was established online by China CDC in 2004 and rodent surveillance sites were adjusted to 40 sites in 22 provinces in 2005. Here we analyzed the surveillance data of both human cases and rodents host during 2006–2012 to examine the epidemic trends of HFRS in recent years in China.

**Methods:**

Records on HFRS human cases and surveillance data of rodents host from 2006 to 2012 were analyzed. Phylogenetic tree based on complete sequence of M segment of 58 virus isolates was constructed and analyzed to make a better understanding of the molecular diversity of hantaviruses in China.

**Results:**

During 2006–2012, a total of 77558 HFRS human cases and 866 deaths were reported with the average annual incidence rate of 0.83 cases/100,000 population and case fatality rate of 1.13%. 84.16% of the total cases were clustered in 9 provinces and mainly reported in spring and autumn-winter seasons. HFRS incidence in males was over 3 times higher than in females and farmers still accounted for the largest proportion. The average density of rodents was relatively stable from 2006 to 2012. *Apodemus agrarius* and *Rattus norvegicus* were predominant in wild field and residential area, respectively. Both hantaviruses carrying and infection rates in rodents had a rapid increase in 2012. Phylogenetic analysis showed that at least six clades of Hantaan virus and five of Seoul virus were prevalent in China.

**Conclusion:**

HFRS in China was still a natural focal disease with relatively high morbidity and fatality and its distribution and epidemic trends had also changed. Surveillance measures, together with prevention and control strategies should be improved and strengthened to reduce HFRS infection in China.

## Background

Hemorrhagic fever with renal syndrome (HFRS), characterized by fever, hemorrhage, kidney damage and hypotension, is an important infectious disease caused by different species of hantaviruses including Hantaan virus (HTNV) [1], Seoul virus (SEOV) [2], Puumala virus (PUUV) [3,4] and Dobrava/Belgrade virus (DOBV/BGDV) [5]. It is reported that the number of HFRS human cases in China accounts for almost 90% of the total cases worldwide [6,7]. Although some prevention and control measures have been performed, HFRS remains a serious public health problem in China with about 20,000-50,000 human cases annually in mainland China [8,9]. From 1981 to 2005, more than 20,000 cases of HFRS occurred annually, with a peak of 115,807 cases and 2569 deaths recorded in 1986 (Additional file 1: Figure S1). Laboratory based national HFRS surveillance system and development of vaccines for hantaviruses (HV) had been initiated for HFRS control and prevention in 1980s in China [10]. In 2004, the National Notifiable Disease Surveillance System (NNDSS) was established online by Chinese Center for Disease Control and Prevention (China CDC) and HFRS cases of the whole country were reported daily through this system.

As HFRS is a rodent-borne disease, rodent surveillance sites were designed to be partially adjusted periodically according to the epidemic situation of hantavirus infection. A total of 48 counties were selected in 30 provinces at beginning in 1984, adjusted to 41 sites in 30 provinces in 1995, and then adjusted to 40 sites in 22 provinces in 2005 [11] (Additional file 2: Figure S2). The major epidemic areas were covered over the whole period of surveillance and epidemiology and geographic distribution of hantavirus infections in rodents host were collected each year.

Here we analyzed the surveillance data of both human cases and rodents host during 2006–2012 to examine the epidemic trends of HFRS in recent years in China. Furthermore, several hantaviruses have been isolated from various rodent species and patients throughout China during these seven years. Preliminary serological characterization of these isolates indicated that HFRS in China was mainly caused by HTNV, SEOV and PUUV. We genetically characterized Chinese strains, along with representative isolates from other countries to obtain basic information on the variability of hantaviruses in China.

## Methods

### Data source

Records on HFRS human cases from 2006 to 2012 were obtained from the NNDSS, an administrative database developed by China CDC. Since 2004, surveillance information, which included number of cases and deaths, demographic characteristics, geographic and temporal distributions have been collected and updated daily. All provinces of the country contributed surveillance data through direct network report. Criteria for reporting cases was referred to the ’Diagnostic criteria for epidemic hemorrhagic fever’ (GB15996—1995 and ws 278—2008) of China.

Surveillance data of rodents host were gained from 22 provinces which collected information including number of mousetraps placed, number of rodents captured, number of lung and blood specimen collected from selected counties, et al. Then laboratory tests were performed and results were reported to related department of China CDC.

### Epidemics of HFRS human cases

Numbers of HFRS cases were counted for each year during 2006–2012. Incidence rates were calculated per 100,000 population by using population estimates based on census of China. Fatality rates were presented as the ratio of dead cases and total cases for each year.

As for geographic and temporal distributions, HFRS cases were sorted by location (province) and time. The average incidence rate of each province was calculated and geographic distribution map of HFRS incidence was drawn using Mapinfo Professional 9.5 Software. Annual incidence from 2006 to 2012 was calculated and plotted to observe annual fluctuations of HFRS in China. Provinces with high incidence were selected and HFRS cases for each month were also analyzed to observe seasonal fluctuations.

Analysis of demographic characteristics was performed for HFRS cases by gender, age group, occupation and region of patients’ residence. 14 age groups with 5-year interval were defined for this study while cases number and fatality rate were used as analysis indicators.

### Surveillance of Rodents host

40 counties in 22 provinces were selected as rodent surveillance sites and over 200,000 mousetraps were placed each year in both spring and autumn-winter seasons. The density of rodents, which was expressed as the ratio of rodents captured and mousetraps placed, was calculated for each year from 2006 to 2012, by seasons, and by type of patients’ residence. Composition of rodent species was classified by surveillance sites and by type of patients’ residence. Geographic maps for species composition were performed using Mapinfo Professional 9.5 Software.

Serological and molecular detection were practiced in samples from rodents host. The lungs of rodents were sliced, then detected by indirect immunofluorescence assay (IFA) to determine HV carrying rates and viral RNA was extracted from lungs for RT-PCR analysis. Serum samples were used for IFA and enzyme-linked immunosorbent assay (ELISA) and HV infection rates were analyzed.

### Phylogenetic analysis

Complete genomic sequences of M segment from 58 hantavirus strains were downloaded from the National Center for Biotechnology Information (NCBI) website and phylogenetic tree was performed using Mega 5.02 software and analyzed with the Maximum Likehood (ML) method with complete deletion of gaps. Bootstrap testing (1000 replicates) was employed. Representative hantaviruses, including HTNV, SEOV, PUUV and DOBV isolates from China, South Korea, Russia, et al. [12-14] and Chinese strains isolated from 2006 to 2012 were included in the phylogenetic analysis.

### Data analysis

All data were analyzed by using standardized Excel spreadsheet software (Microsoft, Redmond, WA, USA) and GraphPad Prism Software (ver. 5.0; GraphPad Software, Inc., San Diego, CA, USA). Geographic maps were performed using Mapinfo Software (ver. Professional 9.5; Mapinfo, USA).

### Ethical consideration

According to the medical research regulation of National Health and Family Planning Commission, China, our work was reviewed and approved by the ethics committee of China CDC, which uses international guidelines to ensure confidentiality, anonymity, and informed consent.

## Results

### Descriptive analysis of HFRS epidemics

From 2006 to 2012, HFRS cases were found in 30 out of 31 provinces of China (excluding Hong Kong, Macao and Taiwan). A total of 77,558 cases (15,098, 11,063, 9,039, 8,745, 9,526, 10,779 and 13,308 cases in 2006–2012, respectively) and 866 deaths were reported with the average annual incidence rate of 0.83 per 100,000, mortality rate of 0.01 per 100,000 and case fatality rate of 1.13% (Table 1).

**Table 1:** Epidemics of HFRS in China and five provinces with highest incidence, 2006-2012

**Years**	**Cases (deaths)**	**Incidence (1/100,000)**	**Mortality (1/100,000)**	**Fatality rate (%)**	**Five provinces with highest HFRS incidence**		**Percentage (%)**
2006	15,098	1.1547	0.0132	1.15	Heilongjiang	3,810	66.95
	(173)				Liaoning	1,961	
					Jilin	1,899	
					Shandong	1,411	
					Shaanxi	1,027	
2007	11,063	0.8416	0.0110	1.31	Heilongjiang	3,087	64.66
	(145)				Jilin	1,043	
					Shaanxi	1,035	
					Shandong	1,002	
					Liaoning	986	
2008	9,039	0.6841	0.0078	1.14	Heilongjiang	1,892	67.21
	(103)				Shaanxi	1,412	
					Shandong	1,154	
					Jilin	880	
					Liaoning	737	
2009	8,745	0.6585	0.0078	1.19	Heilongjiang	1,701	64.69
	(104)				Shaanxi	1,404	
					Shandong	922	
					Jilin	910	
					Liaoning	720	
2010	9,526	0.7137	0.0088	1.24	Shaanxi	2,408	66.89
	(118)				Heilongjiang	1,466	
					Shandong	980	
					Jilin	765	
					Liaoning	753	
2011	10,779	0.8039	0.0089	1.10	Shaanxi	2,605	63.08
	(119)				Heilongjiang	1,576	
					Liaoning	981	
					Shandong	958	
					Jilin	679	
2012	13,308	0.9877	0.0077	0.78	Shaanxi	3,591	66.69
	(104)				Heilongjiang	1,717	
					Shandong	1,696	
					Liaoning	1,126	
					Jilin	745	

### Geographic distribution of HFRS human cases

**Figure 1:**
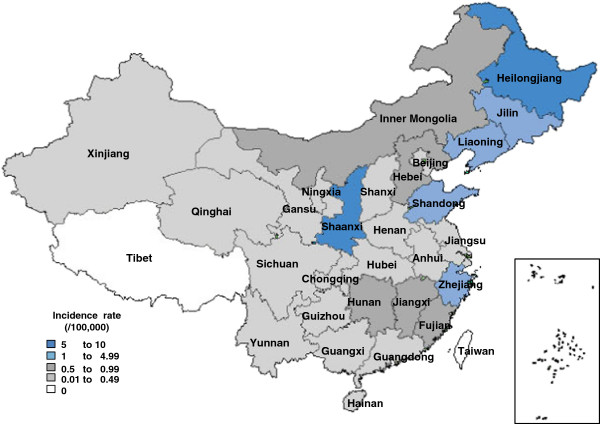
**Geographic distribution of HFRS incidence in China from 2006 to 2012.** HFRS incidence rates in 31 provinces of China were calculated and were divided into five groups representing by different colors.

HFRS human cases were reported in all provinces of China except Tibet during 2006–2012. The top 9 provinces with HFRS cases were Heilongjiang (15,269 cases), Shaanxi (13,482 cases), Shandong (8,123 cases), Liaoning (7,264 cases), Jilin (6,921 cases), Zhejiang (3,971 cases), Hunan (3,966 cases), Hebei (3,269 cases) and Jiangxi (3,008 cases), which accounted for 84.16% of the total number of cases. There were six provinces, of which the average annual incidence rates were greater than 1 per 100,000, that were Heilongjiang (5.70/100,000), Shaanxi (5.14/100,000), Jilin (3.62/100,000), Liaoning (2.41/100,000), Shandong (1.23/100,000) and Zhejiang (1.11/100,000) (Figure 1). HFRS incidence rate in the three provinces of Northeast area showed decreasing trend while in Shaanxi province, incidence was gradually increased during these seven years. From 2006 to 2009, Heilongjiang province ranked first as for HFRS incidence and since 2010, Shaanxi province had ranked first in turns of Heilongjiang (Table 1).

There were totally 25 provinces in China that had reported HFRS deaths from 2006 to 2012, with average annual fatality rate from 0.44%-8.80%, while Shanghai was an exception because it only had one HFRS case in 2012 but dead. The top 3 provinces with HFRS deaths were Heilongjiang (150 deaths), Shandong (122 deaths), Shaanxi (110 deaths) and provinces with highest fatality rate were Chongqing (8.80%), Yunnan (6.73%) and Gansu (5.24%).

### Temporal distribution of HFRS human cases

**Figure 2:**
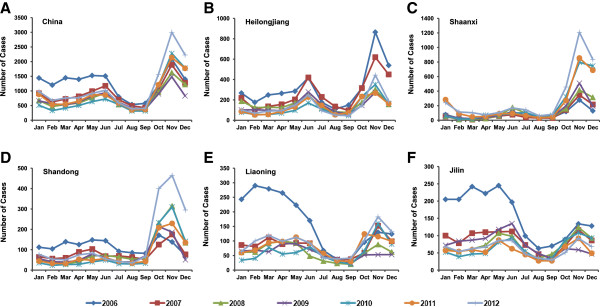
**Temporal distribution of HFRS in China from 2006 to 2012.** HFRS temporal distribution of **A)** the whole country and 5 provinces **B)** Heilongjiang, **C)** Shaanxi, **D)** Shandong, **E)** Liaoning and **F)** Jilin with high incidence was analyzed. The number of cases for each month was calculated and analyzed to observe seasonal fluctuations.

The annual HFRS incidence in China varied from 2006 to 2012, with the highest incidence of 1.15 cases per 100,000 persons in 2006, and the lowest incidence of 0.66 cases per 100,000 persons in 2009. Morbidity had been annually decreasing from 2006 to 2009 but had been increasing since 2010 (Table 1). The temporal trends of HFRS incidence during these 7 years was similar that cases were reported every month, but mainly in spring and autumn-winter seasons during which nearly 65% of HFRS cases were reported (Figure 2A).The top 5 provinces with high incidence (Heilongjiang, Shaanxi, Shandong, Liaoning and Jilin) were selected and HFRS cases for each month were analyzed to observe seasonal fluctuations (Figure 2B-F). As for Heilongjiang, Shaanxi and Shandong provinces, the incidence peak in autumn-winter was higher than in spring season, while Jilin province showed apparent bimodal curves. The autumn-winter peak of 2012 appeared obvious upward trend in Shaanxi, Shandong and Liaoning three provinces, and the spring peak of 2006 was much higher than autumn-winter peak in provinces of Liaoning and Jilin.

### Demographic characteristics of HFRS human cases

**Figure 3:**
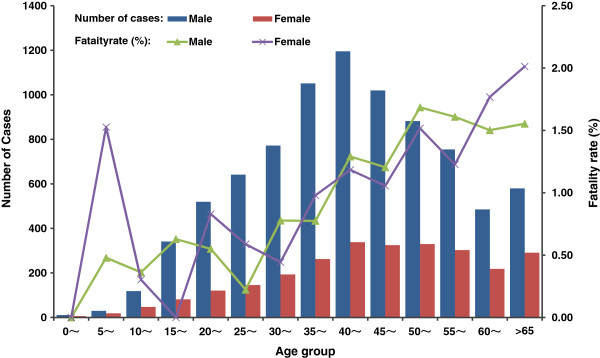
**Demographic characteristics of HFRS in China, 2006–2012.** Numbers of cases and fatality rates of HFRS were analyzed based on gender and age group. 14 age groups with 5-year interval were defined.

Demographic characteristic analysis was stratified by gender, age group, and occupation. Most (75.81%) HFRS cases were for men and the incidence in males were about 3 times higher than in females. Average annual HFRS cases were calculated by gender and 14 age groups, over 90 percent of reported cases were clustered in age groups of 15–65 years while fatality rates increased steadily by advancing age group. Farmers still accounted for the largest proportion (67.80%) of HFRS patients (Figure 3).

### Surveillance of Rodents host

**Figure 4:**
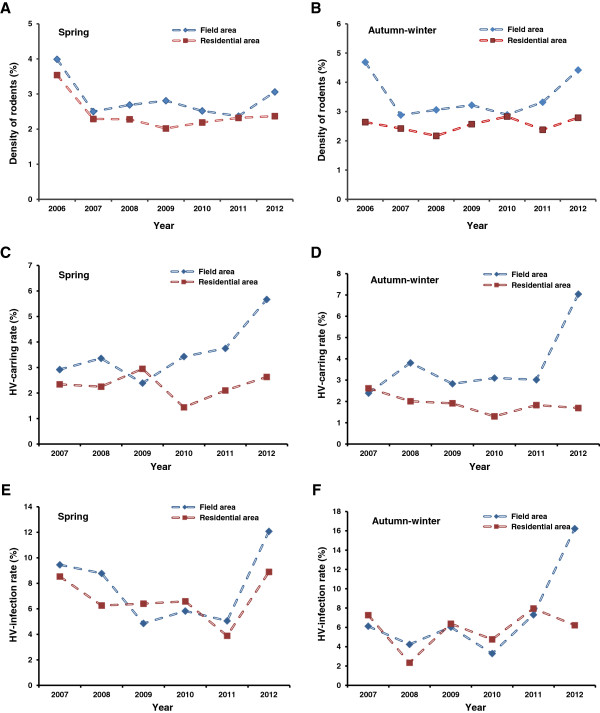
**Surveillance of rodents host, 2006–2012.** The annual **A-B)** average density of rodents, **C-D)** HV carrying rates and **E-F)** infection rates were analyzed by seasons and by type of patients’ residence.

The average density of rodents was relatively stable from 2006 to 2012. Surveillance spots in Heilongjiang, Liaoning, Henan, Inner Mongolia and Jilin were with high density while in some sites of Heilongjiang province, the rodents densities were over 30%. Densities in wild field area were higher than those in residential area, especially in autumn-winter season (Figure 4A-B).Serological and molecular detection were practiced in samples from rodents, showing that the HV carrying rate and infection rate in spring were almost similar to those in autumn-winter season and rapidly increased in 2012 (Figure 4C-D). HV carrying rate in field area was relatively higher than in residential area and infection rate (Figure 4E-F) was much higher in the three provinces of Northeast China.

**Figure 5:**
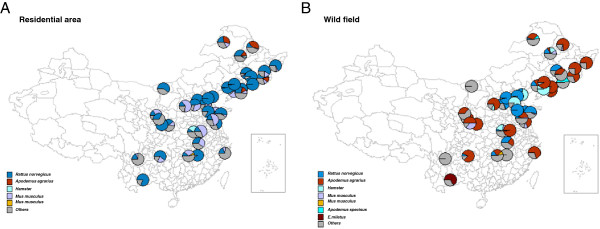
**Composition of rodents species in surveillance sites of China, 2006–2012.** Rodents species was classified by surveillance sites and by type of patients’ residence **A)** residential area; **B)** Wild field. Geographic maps for species composition were performed using Mapinfo 9.5 Software and each pie represented the ratio composition of rodents’ species in one surveillance site.

Analysis of prevailing species indicated that 40 selected surveillance sites contained multiple rodents species. The distribution of dominant species in spring and autumn-winter was similar while in wild field and residential area varied somewhat. *Apodemus agrarius* were dominant in wild field area, followed by *Rattus norvegicus*, while in some surveillance sites there were some other dominant species distributions, such as *Hamsters*, *Mus musculus*, *Flavipectus, E.miletus* and so on (Figure 5B). In residential area, of which the rodents species were less complex, *Rattus norvegicus* was absolutely the dominant rodent, *Mus musculus* also accounted for a certain proportion, other prevailing species including *Hamsters*, *Flavipectus*, et al. (Figure 5A).

### Phylogenetic analysis

**Figure 6:**
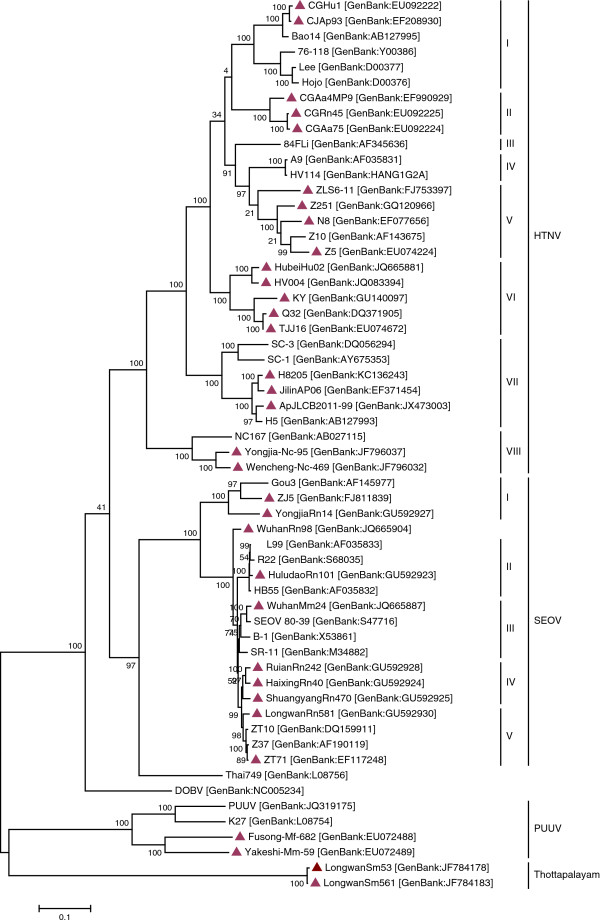
**Phylogenetic trees of hantaviruses based on complete M segment sequences.** The tree was constructed by using the Maximum Likehood (ML) method. Numbers above branches are percentage bootstrap support values for 1000 replicates. Sequences are identified by their strain names, followed by GenBank accession numbers. The scale bars indicate 0.1 substitutions per site and sequences obtained from China during 2006–2012 are labeled with triangle.

To examine the etiology of viruses causing HFRS in China more closely, we examined the genetic characteristics of 58 hantavirus isolates collected from humans or rodents in various geographic locals. As is shown in Figure 6, the branches of the phylogenetic tree for complete M segment formed eight HTN clades (designated subtypes I to VIII) and five SEO clades (designated subtypes I to V). Of the HTN viruses, Chinese strains isolated from 2006 to 2012 belonged to six clades except for subtype III and IV. The genetic diversity of the SEO viruses was lower than that of the HTN viruses. Although there were five subtypes in phylogenetic tree, subtype I to IV appeared to be closely related to one another. And all subtypes consisted of Chinese isolates. Subtype V consisted of Strains Gou3, ZJ5 and Yongjia Rn14, and it seemed distinct from the other SEO viruses in phylogenetic tree.

## Discussion

Our study analyzed the surveillance data of both HFRS human cases and rodents host from 2006–2012 to determine the epidemic trends of HFRS in recent years. Since the year 2004, incidence of HFRS in China has steadily declined year after year till 2009, of which the reported HFRS cases dropped to the lowest level of the past two decades. It is noteworthy that since 2010, HFRS cases have been increasing but still clustered in the Northeast and East China. The reasons for this trend-change are not clear and it may just natural fluctuations in the epidemic process of HFRS. But it still needs to define factors contributed to this, which may affect HFRS incidence and make the control measures more effective.

From 1961–2005, over 20,000 HFRS cases were reported per year while the numbers of cases reported during 2006–2012 were below those reported for previous years. This probably due to the National Expanded Programme on Immunization (EPI) with hantavirus vaccine which have been implemented in selected key endemic sites since the year 2008. Seven provinces that with high incidence rates, including Liaoning, Heilongjiang, Jilin, Shandong, Hebei, Shaanxi and Zhejiang, were firstly chosen as trial areas [15]. And then five other provinces (Jiangxi, Jiangsu, Inner Mongolia, Sichuan and Hubei) had joined in EPI of HV vaccine till June 2010. Besides, public education, rodent control measures and social changing may also contribute to the case.

Compared with previous years, there were little changes in the number of provinces that with high HFRS incidence, but the ranking had varied a lot. From 2006 to 2009, Heilongjiang province ranked first as for incidence rate and since 2010, Shaanxi province had ranked first in turns of Heilongjiang. HFRS incidence showed an obviously decreasing trend in most provinces of China, such as Heilongjiang, Liaoning, Jilin, Hebei and other places, contributing to wide range of vaccination and rodent control [16]. However, in province Shaanxi, Shandong and Jiangxi, HFRS epidemic presented a rebounded trend. This may due to changes of natural or human factors such as changes of rodents habitat caused by abnormal climate, variation of rodents density and HV carrying rate induced by host migration, susceptible population changes infected by non-immune people coming into epidemic areas, et al. [17].

HFRS epidemic during 2006 to 2012 had little changes in demographic characteristics (age, gender and occupation), but the proportion of human cases in age groups of 15 to 60 years had gradually declined from 87.8% in 2006 to 78.8% in 2012, while proportion of incidence in age groups of <15 and >60 years had steadily increased. The target population of EPI project is 15 to 60 years old people in key areas of selected provinces [15,18], although covered the main population with high HFRS incidence, it ignored the protection of persons in other age groups, especially > 60 years. Reported HFRS patients in 60–70 age group, of which people with declined immunity, accounted for 9.8% of the total cases in 2006, and had risen to 13.0% in 2012. The proportion of deaths number in this age group is increased from 10% in 2006 to 20% in 2011, but declined to 14.4% in 2012. Thus it is important to consider vaccine immunization to people aged from 60 to 70, to reduce HFRS incidence and deaths in this population.

HFRS in China were mainly caused by two types of hantaviruses, that were HTNV transmitted by *Apodemus agrarius* and SEOV transmitted by *Rattus norvegicus*. However, because of the limitations of data sources, either records obtained from NNDSS or gained from rodents surveillance sites were insufficient to distinguish the number of HFRS human cases reported or rodents host were caused by Hantaan virus or by Seoul virus. The incidence curve of Apodemus-type HFRS had two peaks (June to July and November to October) while the peak in autumn-winter was much higher than in spring season. The epidemic peak of Rattus-type HFRS was mainly in spring (March to May) [11]. Temporal trend of HFRS from 2006 to 2012 was similar that cases were mainly reported in spring and autumn-winter, but incidence peak of the latter season was higher than that of the former, indicating that HFRS infection in China was mainly caused by *Apodemus*. Analysis on seasonal epidemic in key provinces showed that in recent years the spring incidence peak of different types of epidemic areas presented a downward trend while autumn-winter peak gradually increased, also suggesting that the proportion of Apodemus-type infection in China had been increasing. The density of rodents and HV-carrying rates were important factors affecting HFRS incidence. Surveillance data in selected sites showed that from 2006 to 2012, both rodents density and HV-carrying rates had not changed a lot, indicating that the statuses of HFRS rodents host were relatively stable. However, in some surveillance sites of Heilongjiang, Liaoning and Jilin province, the two factors mentioned above were much higher and in 2012, the HV carrying rates and infection rates had a rapid increase.

To make a better understanding of the molecular diversity of hantaviruses isolated from 2006 to 2012 in China and of their evolutionary relationship with hantaviruses isolated previously or from other HFRS epidemic countries, complete M sequences of 58 isolates obtained from either patients or rodents were selected and analyzed. Phylogenetic tree revealed that at least six clades of HTNV and five of SEOV were prevalent in China during 2006–2012, showing a high degree of genetic diversity. HTN and SEO viruses are found in both humans and rodents in China [19,20] while the genetic variability among HTNV was higher than that among SEOV.

## Conclusion

HFRS were still a natural focal disease with relatively high morbidity and fatality in China. Distribution and epidemic trend of the disease had also changed. Surveillance measures, together with prevention and control strategies should be improved and strengthened to reduce HFRS infection in China.

## Competing interests

There are no competing interests among all authors of this study.

## Authors’ contributions

SZ, SW, WY and ML were involved in surveillance data collection, management and analysis. SZ drafted the manuscript. JL participated in phylogenetic analysis of complete M segment sequences of hantaviruses and QZ participated in laboratory detection of rodents’ samples. ZF manipulated HFRS surveillance. DL is the project leader and was involved in project design, manipulation, data analysis and finalization of the manuscript. All co-authors read and approved the final manuscript.

## Pre-publication history

The pre-publication history for this paper can be accessed here:

http://www.biomedcentral.com/1471-2334/14/384/prepub

## Supplementary Material

Additional file 1: Figure S1 HFRS epidemic from 1950 to 2005. Annual incidence and death of HFRS from 1950 to 2005 in China. The total number of human cases and deaths were graphed for each year according to date of onset and dead. Click here for file

Additional file 2: Figure S2 Rodents surveillance sites of HFRS in China. The pink dots indicated the location of 40 sites in 22 provinces adjusted in the year of 2005. Click here for file
