# Supplementary figures and images for: Epidemic characteristics of hemorrhagic fever with renal syndrome in China, 2006–2012
Source: BMC Infect Dis. 2014 Jul 11;14:384. doi: 10.1186/1471-2334-14-384 (PMC4105051; doi:10.1186/1471-2334-14-384)

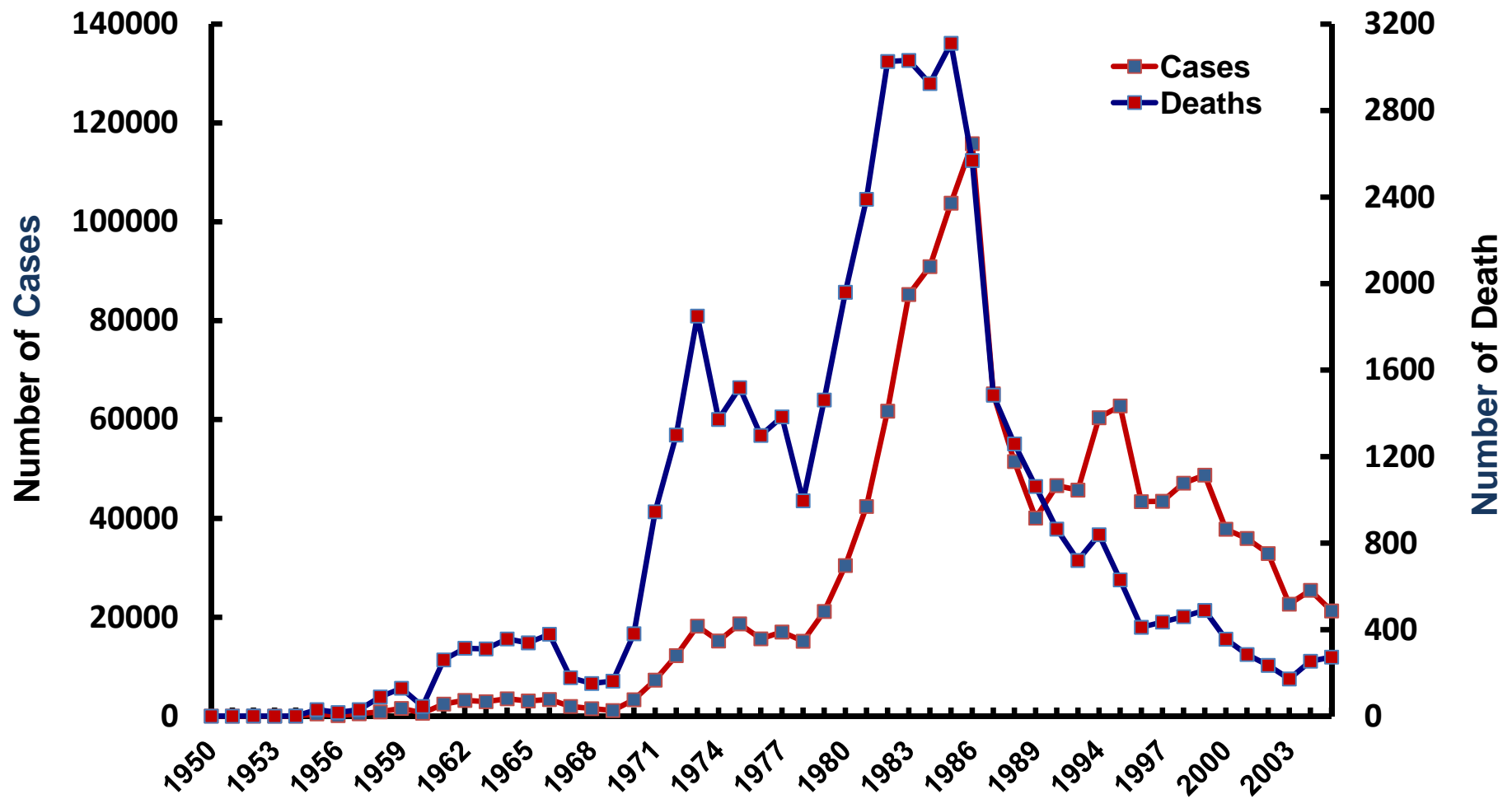

Supplement: Additional file 1: Figure S1 — HFRS epidemic from 1950 to 2005. Annual incidence and death of HFRS from 1950 to 2005 in China. The total number of human cases and deaths were graphed for each year according to date of onset and dead. [file 1471-2334-14-384-S1.pdf]

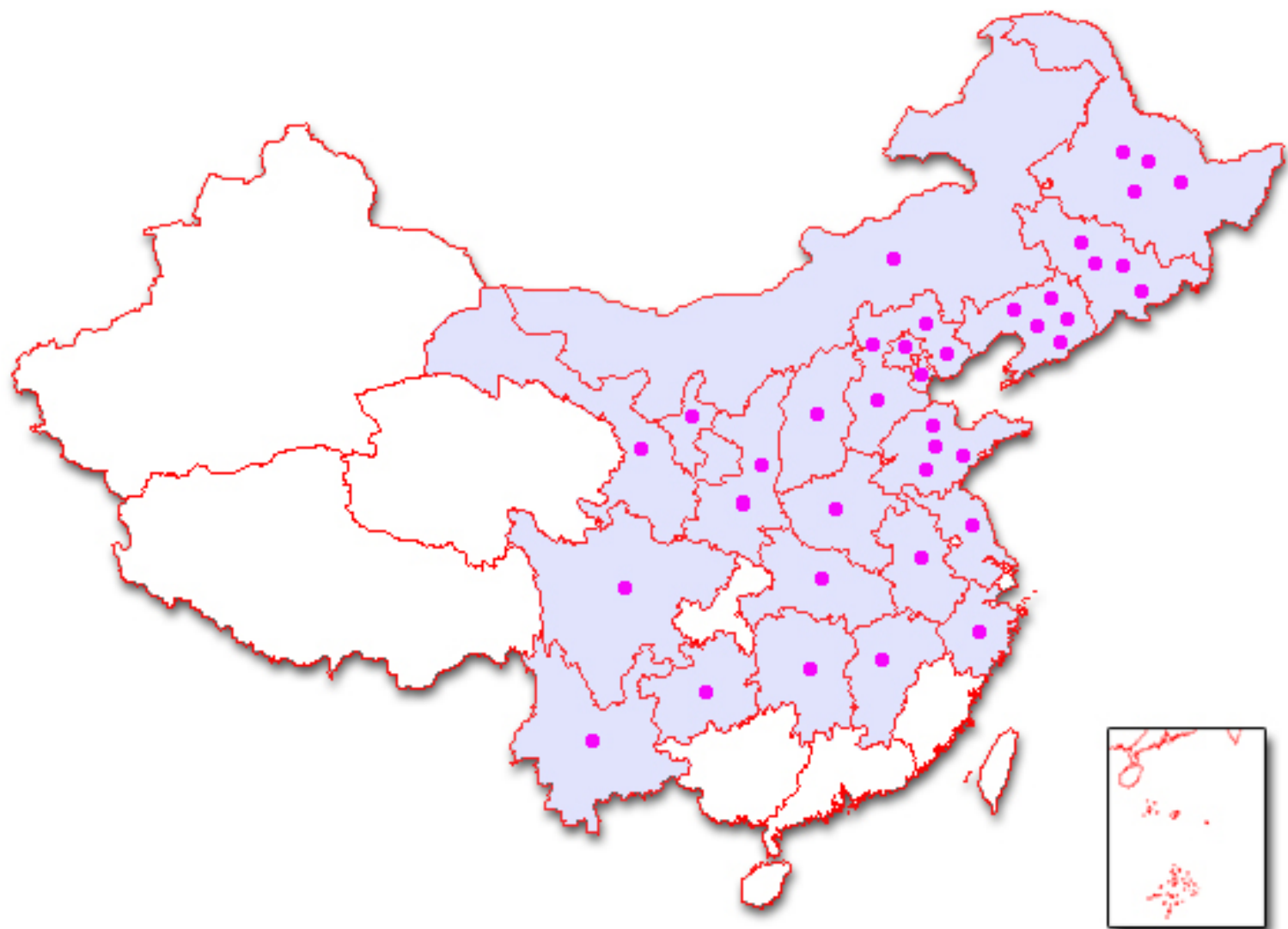

Supplement: Additional file 2: Figure S2 — Rodents surveillance sites of HFRS in China. The pink dots indicated the location of 40 sites in 22 provinces adjusted in the year of 2005. [file 1471-2334-14-384-S2.pdf]
